# Flavonoid Naringenin Alleviates Short-Term Osmotic and Salinity Stresses Through Regulating Photosynthetic Machinery and Chloroplastic Antioxidant Metabolism in *Phaseolus vulgaris*

**DOI:** 10.3389/fpls.2020.00682

**Published:** 2020-06-03

**Authors:** Evren Yildiztugay, Ceyda Ozfidan-Konakci, Mustafa Kucukoduk, Ismail Turkan

**Affiliations:** ^1^Department of Biotechnology, Faculty of Science, Selcuk University, Konya, Turkey; ^2^Department of Molecular Biology and Genetics, Faculty of Science, Necmettin Erbakan University, Konya, Turkey; ^3^Department of Biology, Faculty of Science, Selcuk University, Konya, Turkey; ^4^Department of Biology, Faculty of Science, Ege University, Bornova, Turkey

**Keywords:** antioxidant enzymes, chloroplast isolation, naringenin, photosynthetic efficiency, stress

## Abstract

The current study was conducted to demonstrate the possible roles of exogenously applied flavonoid naringenin (Nar) on the efficiency of PSII photochemistry and the responses of chloroplastic antioxidant of salt and osmotic-stressed *Phaseolus vulgaris* (*cv. Yunus90)*. For this aim, plants were grown in a hydroponic culture and were treated with Nar (0.1 mM and 0.4 mM) alone or in a combination with salt (100 mM NaCl) and/or osmotic (10% Polyethylene glycol, −0.54 MPa). Both caused a reduction in water content (RWC), osmotic potential (Ψ_Π_), chlorophyll fluorescence (F_v_/F_m_), and potential photochemical efficiency (F_v_/F_o_). Nar reversed the changes on these parameters. The phenomenological fluxes (TR_o_/CS and ET_o_/CS) altered by stress were induced by Nar and Nar led to a notable increase in the performance index (PI_ABS_) and the capacity of light reaction [ΦP_o_/(1-ΦP_o_)]. Besides, Nar-applied plants exhibited higher specific fluxes values [ABS/RC, ET_o_/RC, and ΨE_o_/(1-ΨE_o_)] and decreasing controlled dissipation of energy (DI_o_/CS_o_ and DI_o_/RC). The transcripts levels of psbA and psbD were lowered in stress-treated bean but upregulated in Nar-treated plants after stress exposure. Nar also alleviated the changes on gas exchange parameters [carbon assimilation rate (A), stomatal conductance (g_s_), intercellular CO_2_ concentrations (C_i_), transpiration rate (E), and stomatal limitation (L_s_)]. By regulating the antioxidant metabolism of the isolated chloroplasts, Nar was able to control the toxic levels of hydrogen peroxide (H_2_O_2_) and TBARS (lipid peroxidation) produced by stresses. Chloroplastic superoxide dismutase (SOD) activity reduced by stresses was increased by Nar. In response to NaCl, Nar increased the activities of ascorbate peroxidase (APX), glutathione reductase (GR), monodehydroascorbate reductase (MDHAR), and dehydroascorbate reductase (DHAR), as well as peroxidase (POX). Nar protected the bean chloroplasts by minimizing disturbances caused by NaCl exposure via the ascorbate (AsA) and glutathione (GSH) redox-based systems. Under Nar plus PEG, Nar maintained the AsA regeneration by the induction of MDHAR and DHAR, but not GSH recycling by virtue of no induction in GR activity and the reduction in GSH/GSSG and GSH redox state. Based on these advances, Nar protected in bean chloroplasts by minimizing disturbances caused by NaCl or PEG exposure via the AsA or GSH redox-based systems and POX activity.

## Introduction

Plants are simultaneously subjected to the combination of salinity and osmotic stresses rather than the effects of an individual stress. Plants have common defense responses against these stresses. Photosynthesis is inhibited by decreasing intercellular CO_2_ concentrations depending on stomatal closure under both salt and osmotic stresses (Cheeseman, 2013). Chloroplasts, one of the cell compartments, have a central role in cell pathways such as biosynthesis of aromatic amino acids, fatty acids and carotenoids, and sulfate and nitrogen assimilation, as well as photosynthesis and are sensitive to stress conditions. Some of symptoms triggered by stress in plants is associated with the process occurred in chloroplasts. Due to the highly energetic reactions of photosynthesis, the reduction of molecular oxygen generates toxic reactive oxygen species (ROS), which interacts with essential and important molecules such as DNA, photosynthetic pigments, and proteins (Miller et al., 2010; Flowers et al., 2015). The accumulation of ROS causes the photoinhibition of photosystem I (PSI), the disruption in the structure of photosynthetic pigments, the inactivation of the elongation factor of D1 protein, the inhibition in the repair of photosystem II (PSII) and the decline of rubisco activity (Acosta-Motos et al., 2017; Bose et al., 2017). Stress treatments easily occur the damage in the reaction centers (RCs) of PSII (Strauss et al., 2006). The data on the photosynthetic performance is provided with the phenomenological and biophysical parameters in plants (Gururani et al., 2015). The responses of photosynthetic apparatus with these parameters are evaluated through OJIP fluorescence transients (called a JIP test). The OJIP test was developed by Strasser et al. (1995), and the parameters are calculated using the generated chlorophyll fluorescence induction curve according to the JIP-test method (Zeliou et al., 2009). The JIP test shows the measurement of several phenological and biophysical expressions of PSII including the fluxes of absorption, trapping, and electron transport (Strasser et al., 2000).

Cell detoxification mechanisms against ROS accumulation include the induced gene expression of enzymatic antioxidants including superoxide dismutase (SOD), peroxidase (POX), and ascorbate peroxidase (APX), glutathione reductase (GR), monodehydroascorbate reductase (MDHAR), dehydroascorbate reductase (DHAR) that are related to AsA-GSH cycle (Asada-Halliwell pathway) (Dai et al., 2012; Uzilday et al., 2012; Carvalho et al., 2015). APX, along with the oxidation of AsA, generates monodehydroascorbate (MDHA). MDHA is spontaneously turned into the oxidized state (dehydroascorbate, DHA). MDHA and DHA are reduced to AsA by the activities of NADPH-dependent MDHAR and GSH-dependent DHAR, respectively. As well as AsA, GSH has important roles to cope with the damage caused by ROS (Batth et al., 2017). To maintain of GSH pool, oxidized glutathione (GSSG) is reduced by GR via the consumption of NADPH (de Sousa et al., 2016).

Flavonoids are small molecular secondary metabolites synthesized by plants. The phenylpropanoid pathway is responsible for the synthesis of flavonoids (D’Amelia et al., 2018). After stress exposure to plants, the biosynthesis of flavonoids is induced. The flavonoids diminished the negative effects of stressful conditions by inhibition of ROS-generating enzymes such as lipoxygenase and xanthine oxidase, the chelation of transition metal ions, and the scavenging activity as an antioxidant agent (Baskar et al., 2018). Their eliminating feature against ROS comes from their catechol group in the B-ring of the flavonoid skeleton. They accumulate in mesophyll cells, vacuole and chloroplasts and stabilize the chloroplast membrane against stress (Martinez et al., 2016). Depending on the location of the flavonoids, some reports suggested them as an important component in photoprotection under stress (Agati and Tattini, 2010). For example, chloroplast-localized flavonoids reduced the ROS generation in excess light irradiance-treated *Phyllirea latifolia* (Agati et al., 2002). Also, flavonoids within chloroplasts protect the function of the membrane during cellular dehydration (Inoue, 2011). Many data revealed that there is an interaction between the levels of induced flavonoids and antioxidant action under different stress treatments such as ozone, heat and salinity (Gondor et al., 2016; Martinez et al., 2016; Pheomphun et al., 2019). Mahajan and Yadav (2013) found that exogenous flavonoid quercetin and epicatechin regulates the antioxidant enzymes at transcriptional levels in tobacco seedlings. As well as abiotic factors, upon exposure to biotic stress conditions, bacterial or fungal-mediated infection is inhibited by flavanols (such as myricetin) and anthocyanins (such as delphinidin) (Karageorgou and Manetas, 2006).

Naringenin (Nar) is a flavonoid belonging to flavanones subclass. It is widely distributed in several citrus fruits, bergamot, tomatoes, and other fruits, being found in its glycosides form (mainly naringin) as well. The chemical name of Nar is 2,3-dihydro-5,7-dihydroxy-2-(4-hydroxyphenyl)-4H-1-benzopyran-4-one (Salehi et al., 2019). Nar is derived from the hydrolysis of glycone forms of this flavanone, such as naringin or narirutin. The effects of Nar have been one of the immense study topics in animal systems (Salehi et al., 2019). However, the number of researches on its possible roles on plant growth, metabolism, and stress responses in plants is rather scanty. In several of those, Nar has been reported to suppress the growth of annual plant species, acting as an allelochemical. Similarly, Nar has been reported to cause a decrease in the growth of *Arabidopsis thaliana* (Hernandez and Munne-Bosch, 2012). This inhibitory effect of Nar was attributed at least to some extent, through impaired auxin transport. Deng et al. (2004) suggested that it exert its inhibitory effect by inhibiting the activity of the key enzyme of the phenylpropanoid pathway, 4-coumarate: CoA ligase, whereas Bido et al. (2010) assumed that the action site of naringenin may be related to other enzymes working at later steps of the phenylpropanoid pathway, such as cell wall–bound POX or, perhaps, cinnamyl alcohol dehydrogenase. Hence, the mode of action of naringenin still remains an open question in plant systems. However, the information about the interaction flavonoids including Nar with the activity of antioxidants localized in chloroplasts under individual or combined-treated stress treatments is unclear. Besides, hardly any data is available about the influence of exogenous applied flavonoid on quantum efficiencies and phenomenological energy fluxes indicating the vitality of PSII in photosynthetic machinery after stress exposure. In this regard, after chloroplast isolation of sampling groups in bean (*Phaseolus vulgaris* L.) leaves, we have focused on explanation the effects of exogenously applied Nar in five steps: (i) the effects of Nar on water content and osmotic potential under salt and/or osmotic stresses; (ii) the effects of Nar on gas exchange parameters such as carbon assimilation rate, transpiration rate, stomatal conductance and stomatal limitation; (iii) the determination the effects of Nar on the photochemical reactions and fluorescence transients and, on the expression levels of genes encoding the major extrinsic proteins of PSII such as psbA and psbD; (iv) the effects of Nar on the responses of antioxidant in chloroplasts of stress-treated plants; and (v) the effects of Nar on ROS content and lipid peroxidation in chloroplasts.

## Materials and Methods

### Plant Material

Common bean seeds (*P. vulgaris cv. Yunus90*) were obtained from the Bahri Dagdas International Agricultural Research Institute, Turkey. The procedures of germination and growth were cited from Yildiztugay et al. (2014).

### The Applications of Stress and Naringenin and the Sampling

For naringenin (Nar; 0.1 mM and 0.4 mM) and salt (NaCl, 100 mM)/osmotic Polyethylene glycol, 10% PEG6000, −0.54 MPa (Hellal et al., 2018) stress treatments, it was prepared by being dissolved in Hoagland solution and was added to the growth medium at the stage of 21 days old. An experiment was designed as twelve groups and was listed in Supplementary Table S1. The toxic levels of NaCl and PEG were chosen base on the study of Kurniasih et al. (2016) and Shatpathy et al. (2018), respectively. For determination of Nar application, the doses of Nar were selected as 0.1 mM and 0.4 mM according to Liu et al. (2012). Plants were harvested after 72 hours (h) of treatment.

### Determination of Water Content and Osmotic Potential

After harvest, six leaves were obtained and their fresh weight (FW) was determined. The leaves were floated on de-ionized water for 6 h and the turgid tissue was blotted dry prior to determining turgid weight (TW). Dry weight (DW) was determined after oven drying at 70°C. The leaf relative water content (RWC) was calculated by the following formula (Smart and Bingham, 1974):

RWC(%)=[(FW-DW)/(TW-DW)]×100

Leaves were extracted by crushing the material with a glass rod. Leaf osmotic potential (Ψ_Pi_) was measured by Vapro Vapor pressure Osmometer 5600. Ψ_Pi_ was converted to MPa according to Santa-Cruz et al. (2002) by multiplying by a coefficient of 2.408 × 10^–3^.

### Determination of Photosynthetic Efficiency and OJIP Analysis

A portable fluorometer (Handy PEA, Hansatech Instruments Ltd., Norfolk, United Kingdom) was used to determine the maximal quantum yield of PSII photochemistry (F_v_/F_m_), physiological state of the photosynthetic apparatus (F_o_/F_m_) and potential photochemical efficiency (F_v_/F_o_). Many parameters showing the structure and function of photosynthetic apparatus were detected by Handy PEA (Plant Efficiency Analyzer, Hansatech Instruments Ltd). The measurement was defined in Table 1 and the radar plot included the average values of the photosynthetic parameters of treatments groups in bean plants.

**TABLE 1:** The abbreviations and definitions of terms used by the JIP-test.

**Abbreviations**	**Definitions**
Area	Total complementary area between the fluorescence induction curve and F_m_
RC	Reaction center
CS	Cross section or measured area of sample
F_o_	Minimal fluorescence of dark-adapted leaves
F_m_	Maximal fluorescence of dark-adapted leaves
F_v_	Variable chlorophyll fluorescence (F_m_-F_o_)
ET	Electron transfer
F_v_/F_m_	Maximum quantum yield of primary photochemistry of PSII
F_o_/F_m_ or φDo	Quantum yield of absorbed photons for electron transport
F_v_/F_o_	Efficiency of the water-splitting complex on the donor side of PSII
ET_o_/CS_o_	Electron transport flux per cross section (CS) at *t* = 0
TR_o_/CS_o_	Trapping per excited cross section
DI_o_/CS_o_	Dissipated energy flux per cross section (CS) at *t* = 0
ABS/RC	Absorption flux (of antenna chlorophylls) per RC
ET_o_/RC	Electron transport flux (further than Q_A_^–^) per RC
TR_o_/RC	Trapped energy flux (leading to Q_A_ reduction) per RC
ΦP_o_/(1-ΦP_o_)	Q_A_-reducing RCs per PSII antenna chlorophyll
ΨE_o_/(1-ΨE_o_)	the efficiency with which a trapped exciton transfers an electron to the photosynthetic ET chain
γRC/(1-γRC)	The fraction of PSII chlorophyll a molecule that function as reaction centers
DI_o_/RC	Dissipated energy flux per reaction center
PI_ABS_	Performance index (potential) for energy conservation from exciton to the reduction of intersystem electron acceptors
PI_total_	Performance index (potential) for energy conservation from exciton to the reduction of PSI and acceptors

### Gene Expression Analysis

RNA isolation was completed given by Ozgur et al. (2015). The nucleic acid concentrations of total RNA and cDNA were measured by a Multiskan Go (Thermo Fisher Scientific, Waltham, MA, United States). To detect relative gene expression for each group, the threshold cycle value was normalized the actin and each sample was evaluated with three replications. The primer sequences and qRT-PCR conditions are given in Supplementary Table S2.

### Determination of Gas Exchange Parameters

Carbon assimilation rate (A), stomatal conductance (g_s_), intercellular CO_2_ concentration (C_i_) and transpiration rate (E) were detected with a portable gas exchange system (LCpro^+^; ADC, Hoddesdon, United Kingdom). The stomatal limitation value (L_s_) was calculated as 1 – C_i_/C_a_ (Ma et al., 2011).

### The Isolation Procedure for Chloroplasts of *Phaseolus vulgaris*

The leaves were homogenized using a blender in isolation buffer containing 0.1 M Tris–HCl (pH 7⋅8), 0.3 M sorbitol, 5 mM MgCl_2_, 10 mM NaCl, 0.1% bovine serum albumin (BSA). The homogenate was filtered through four layers of cheesecloth, and the filtrate was centrifuged at 1000 × *g* for 6 min at 4°C. The supernatant was discarded, and the pellet was resuspended in the isolation buffer. Resuspended chloroplasts were overlaid on a 40% Percoll solution and centrifuged at 1700 × *g* for 7 min at 4°C. Intact chloroplasts were obtained after this centrifugation as a pellet which was resuspended again in the isolation buffer without BSA. Intactness of the chloroplasts was determined by using a ferricyanide reduction test (Lilley et al., 1975). For analysis of enzyme activity and for the native activity gels, chloroplasts were lysed with a lysis solution (10 mM HEPES-KOH (pH 7.2), 0.1 mM EDTA, 1 mM MgCl_2_, 0.1% Triton-X100) for 1 h at 4°C. Where APX was estimated, 5 mM ascorbate was added into the isolation buffer.

### Determination of Isozyme and/or Enzyme Compositions

The total soluble protein content was analyzed (Bradford, 1976). The electrophoretic separation was detected on non-denaturing polyacrylamide miniature slab gels (8 cm × 10 cm) using the Mini PROTEAN Tetra Cell electrophoresis (Bio-Rad). Due to the high number of treatment groups and the low number of wells in the electrophoresis apparatus, the groups were divided into two as (i) C, Nar1, Nar2, S, S + Nar1, S + Nar2; and (ii) D, S, D + Nar1, D + Nar2, SD, SD + Nar1, SD + Nar2 and the gels were loaded to two different running modules at the same time. Samples were exposed to non-denaturing polyacrylamide gel electrophoresis (PAGE) as shown by Laemmli (1970). Chloroplastic SOD (EC 1.15.1.1) activity assay was based on the method of Beauchamp and Fridovich (1971). SOD isozyme activity was detected by staining with riboflavin and nitroblue tetrazolium. SOD isozyme patterns were determined by incubating the gels with 5 mM H_2_O_2_ to inhibit both Cu/Zn-SOD and Fe-SOD, or with 5 mM KCN to inhibit only Cu/Zn-SOD. Before staining of SOD activity, the control group was preincubated with 50 mM potassium phosphate buffer (pH 7.8) alone, buffer plus H_2_O_2_, or buffer plus KCN. POX isozymes were detected according to Seevers et al. (1971). The POX (EC 1.11.1.7) activity was done according to the procedure given by Herzog and Fahimi (1973). Electrophoretic APX separation was performed according to Mittler and Zilinskas (1993). Glutathione S-transferase (GST, EC: 2.5.1.18) activity was calculated following the method of Hossain et al. (2006). For GST isozyme activity, the method of Ricci et al. (1984) was used. NADPH oxidase (NOX) isozymes were identified by NBT reduction method as described by Sagi and Fluhr (2001). The samples containing 40 mg protein was loaded per lane. Total NOX (EC 1.6.3.1) activity was measured according to Jiang and Zhang (2002). The assay medium contained 50 mM Tris–HCl buffer, 0.5 mM XTT, 100 mM NADPH.Na_4_ and 20 mg of protein sample. After addition of NADPH, XTT reduction was followed at 470 nm. Activity was calculated using the extinction coefficient, 2.16 × 10^4^ M^–1^ cm^–1^. One unit of NOX was defined as 1 nmol ml^–1^ XTT oxidized min^–1^.

### Determination of Enzyme Activity Related to AsA-GSH Cycle

Chloroplastic activities of APX (EC 1.11.1.11), GR (EC 1.6.4.2), monodehydroascorbate reductase (MDHAR; EC 1.6.5.4), dehydroascorbate reductase (DHAR; EC 1.8.5.1) were detected according to Foyer and Halliwell (1976); Nakano and Asada (1981), Dalton et al. (1986), and Miyake and Asada (1992), respectively. Total and reduced contents of ascorbate (AsA) were determined according to the method of Dutilleul et al. (2003). The oxidized form of ascorbate (DHA, dehydroascorbate) was calculated using the formula DHA = Total AsA - Reduced AsA. Glutathione (GSH) was assayed according to Paradiso et al. (2008). Oxidized glutathione (GSSG) was determined after removal of GSH by 2-vinylpyridine derivatization.

Gels stained with chloroplastic SOD, POX, APX, NOX, and GST activities were photographed with the Gel Doc XR^+^ System and then analyzed with Image Lab software v4.0.1 (Bio-Rad, Hercules, CA, United States). Activities of isoenzymes (0.5 units of SOD and 0.2 units of POX) were measured according to the known standard amounts.

### Determination of H_2_O_2_ Content and Lipid Peroxidation Levels

H_2_O_2_ was determined according to Cheeseman (2006) using eFOX reagent. This modified ferrous ammonium sulphate/xylenol orange (FOX) assay was used due to its sensitivity, stability, and adaptability to a large number of samples. In this assay, 1% ethanol is added to the reagent, which increases its sensitivity to H_2_O_2_ by 50% (i.e., eFOX). Extraction was carried out using ice-cold acetone containing 25 mM H_2_SO_4_ for intact chloroplasts. Samples were then centrifuged for 5 min at 3000 × *g* at 4°C. eFOX reagent [950 μL of 250 μM ferrous ammonium sulfate, 100 μM xylenol orange, 100 μM sorbitol, 1% ethanol (v/v)] was used for 50 μL of supernatant. Reaction mixtures were incubated at room temperature for 30 min and then absorbance at 550 and 800 nm was measured. H_2_O_2_ concentrations were calculated using a standard curve prepared with known concentrations of H_2_O_2_.

Lipid peroxidation (thiobarbituric acid reactive substances (TBARS) content) was determined according to Rao and Sresty (2000). TBARS concentration was calculated from the absorbance at 532 nm, and measurements were corrected for non-specific turbidity by subtracting the absorbance at 600 nm. The concentration of TBARS was calculated using an extinction coefficient of 155 mM^–1^ cm^–1^.

### Statistical Analysis

The experiments were repeated thrice independently, and each data point was the mean of six replicates. All data obtained were subjected to a one-way analysis of variance (ANOVA). Statistical analysis of the values was performed by using SPSS 20.0. Tukey’s post-test was used to compare the treatment groups. Comparisons with *p* < 0.05 were considered significantly different. In all figures, the error bars represent standard errors of the means.

## Results

### The Effects of Nar on Physiological Parameters in Response to Stress

**FIGURE 1:**
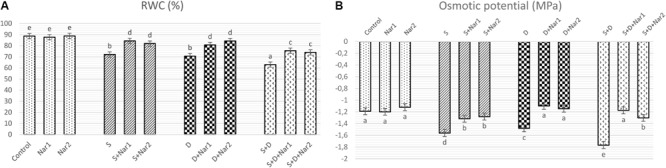
The effects of exogenously applied Nar (Nar1, 0.1 mM and Nar2, 0.4 mM) on water status in bean leaves under alone or combination of salt (S, 100 mM NaCl) and PEG-induced osmotic (D, 10% PEG6000) stress. **(A)** the relative water content (RWC), **(B)** the osmotic potential (Ψ_Π_). For each group, vertical bars indicate ± SE and the different lowercase letters are significantly different (*p* < 0.05) values according to the Tukey test.

Figure 1A shows that there was a significant decrement in RWC under the alone or the combined treatments of stresses. There was a maximum reduction in the combination form of stresses (NaCl plus PEG) by 1.4-fold. The presence of Nar provided the high levels of RWC under stress-treated plants. On the other hand, Nar alone did not affect RWC compared to the control group.

Salinity and osmotic stresses led to a considerable decrease in Ψ_Π_ of bean (Figure 1B). The decreased values of Ψ_Π_ were reversed by exogenously applied Nar in response to stress. Similar to the results of RWC, no effect was created on Ψ_Π_ through Nar alone under control conditions.

### The Effects of Nar on Photosynthetic Efficiency in Response to Stress

**FIGURE 2:**
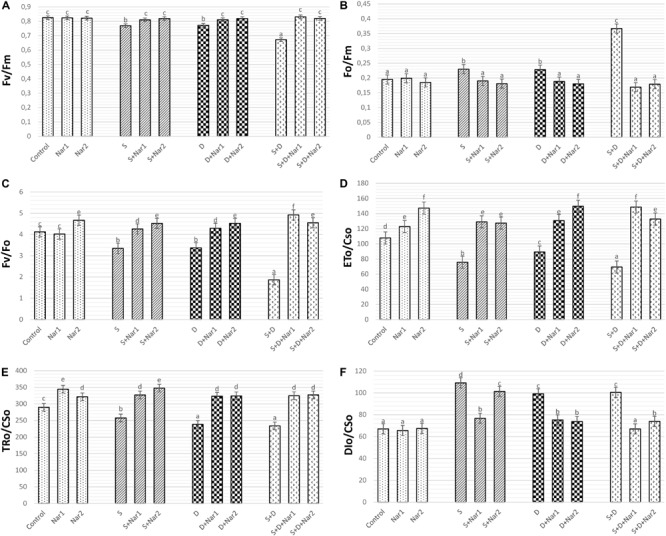
The effects of exogenously applied Nar (Nar1, 0.1 mM and Nar2, 0.4 mM) on the photosynthetic machinery and the phenomenological energy fluxes in bean leaves under alone or combination of salt (S, 100 mM NaCl) and PEG-induced osmotic (D, 10% PEG6000) stress. **(A)** the maximal quantum yield of PSII photochemistry (F_v_/F_m_), **(B)** the physiological state of the photosynthetic apparatus (F_o_/F_m_), **(C)** the potential photochemical efficiency (F_v_/F_o_), **(D)** the electron transport flux per cross section (ET_o_/CS_o_), **(E)** the energy flux trapped per cross section (CS) (TR_o_/CS_o_), **(F)** The dissipated energy flux per cross section (DI_o_/CS_o_). For each group, vertical bars indicate ± SE and the different lowercase letters are significantly different (*p* < 0.05) values according to the Tukey test.

Figures 2A–C revealed that both stress treatments significantly inhibited F_v_/F_m_ and F_v_/F_o_ of bean leaves. The lowest levels of F_v_/F_m_ and F_v_/F_o_ were at the combination form of stresses (by 18.5 and 54.6% declines, respectively). However, stress caused an induction in F_o_/F_m_ and this effect induced by stress was more noticeable at NaCl + PEG group by 88.2% increase (Figure 2B). When bean plants were treated with Nar applications under stress, remarkable responses were created on F_v_/F_m_, F_o_/F_m_, and F_v_/F_o_ levels. The results for these parameters were close to control group or higher than that of one. However, no effect on F_v_/F_m_, F_o_/F_m_, and F_v_/F_o_ was detected after the solo applications of Nar (except for Nar2 on F_v_/F_o_).

The phenomenological energy fluxes are presented in Figures 2D–F. Stress treatments resulted in a decline in ET_o_/CS_o_ (Figure 2D), and TR_o_/CS_o_ (Figure 2E), but the increment in DI_o_/CS_o_ was observed at NaCl and/or PEG stress (Figure 2F). On the other hand, there was the opposite changes in all these parameters of Nar-treated bean plants, as compared to the stress alone. Similar to stress + Nar groups, Nar applications under control conditions promoted the levels of ET_o_/CS_o_ and TR_o_/CS_o_.

### The Effects of Nar on Photosynthetic Machinery in Response to Stress

**FIGURE 3:**
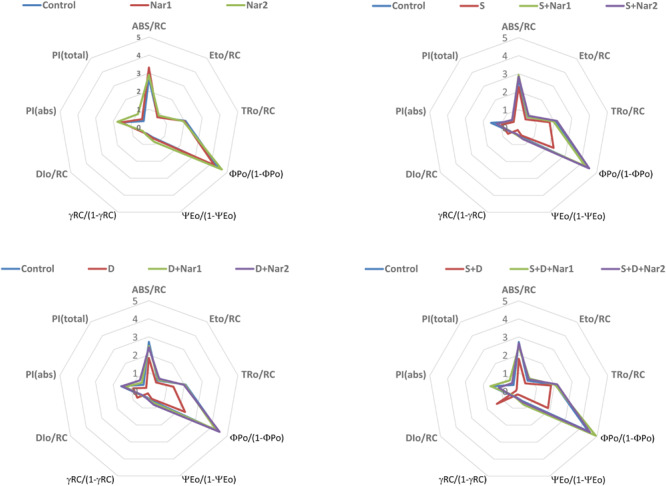
The effects of exogenously applied Nar (Nar1, 0.1 mM and Nar2, 0.4 mM) on the radar plot with a series parameter derived from JIP-test analyses of the experimental fluorescence OJIP transients in bean leaves under alone or combination of salt (S, 100 mM NaCl) and PEG-induced osmotic (D, 10% PEG6000) stress. ABS/RC, average absorption per active reaction center; ET_o_/RC, electron transport flux per active reaction centers; TR_o_/RC, flux or exciton trapped per active reaction center; ΦP_o_/(1-ΦP_o_), Q_A_-reducing RCs per PSII antenna chlorophyll; ΨE_o_/(1-ΨE_o_), the efficiency with which a trapped exciton transfers an electron to the photosynthetic electron transfer chain; γRC/(1-γRC), Q_A_- reducing reaction centers per PSII antenna chlorophyll; DI_o_/RC, ratio of total dissipation to the amount of active reaction center; PI_ABS_, performance index based on the absorption of light energy; PI_total_, performance index (potential) for energy conservation from exciton to the reduction of PSI and acceptors.

Figure 3 showed the values of photosynthetic parameters in all the treatment groups as a radar plot. NaCl or PEG and their combined form caused the similar responses in photosynthetic machinery. While, the specific energy fluxes in thylakoid membranes per reaction centers of sample (ABS/RC, ET_o_/RC, and TR_o_/RC) decreased in chloroplasts of bean with stress, this effect was reversed by Nar applications. These parameters were similar to the control group or increased in Nar-treated plants.

NaCl and/or PEG caused a decrease in the efficiency of light reaction [ΦP_o_/(1-ΦP_o_)] and the rate of biochemical reaction [ΨE_o_/(1-ΨE_o_)] and γRC/(1-γRC), but Nar applications resulted in an increase in these parameters. Interestingly, stress resulted in an increment in energy dissipation (DI_o_/RC) of bean chloroplasts. However, these values were reversed by the Nar applications. Depending on the changes of these parameters [ΦPo/(1-ΦPo), ΨEo/(1-ΨEo) and γRC/(1-γRC)], stress lowered the performance index detected on energy absorption of chloroplasts (PI_ABS_ and PI_total_) and the reduction rate was lower in PEG-treated plants than that of the NaCl ones. Also, the lowest reduction was under the combined stress treatments. Both Nar applications alone were provided the high index levels.

### The Effects of Nar on the Transcription Levels of psbA and psbD in Response to Stress

**FIGURE 4:**
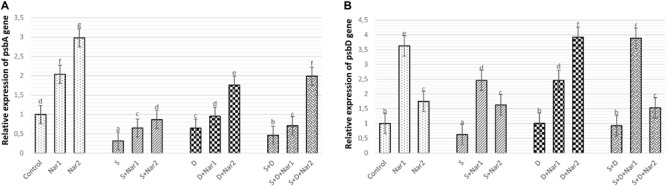
The effects of exogenously applied Nar (Nar1, 0.1 mM and Nar2, 0.4 mM) on the relative expression of genes encoding reaction center core proteins in photosystem of bean chloroplasts under alone or combination of salt (S, 100 mM NaCl) and PEG-induced osmotic (D, 10% PEG6000) stress. **(A)** the relative expression of psbA, **(B)** the relative expression of psbD. For each group, vertical bars indicate ± SE and the different lowercase letters are significantly different (*p* < 0.05) values according to the Tukey test.

Stress had considerable effect on the relative transcription of the psbA gene in bean leaves, which was a maximum reduction in NaCl-treated plants (Figure 4A). Both Nar alone and Nar together with stress assuaged the stress-triggered reduction in psbA transcription compared to that of the control and stress alone group, respectively. The transcription levels of this gene were noticeably increased by a 0.4 mM Nar application under stress or non-stress.

While NaCl-induced salinity suppressed the gene expression of psbD to its lowest levels (by 37.5% decrease), there was no significant difference among the control plants, PEG- and NaCl + PEG-treated plants (Figure 4B). Exogenously applied Nar to stressed plants upregulated the psbD gene to a level above that observed in the stress-treated alone. The psbD gene detected the highest upregulation after S + D + Nar1 by 4.1-fold. In terms of the transcript levels of psbD gene, similar response was observed at Nar alone as compared to the control group.

### The Effects of Nar on Gas Exchange in Response to Stress

**FIGURE 5:**
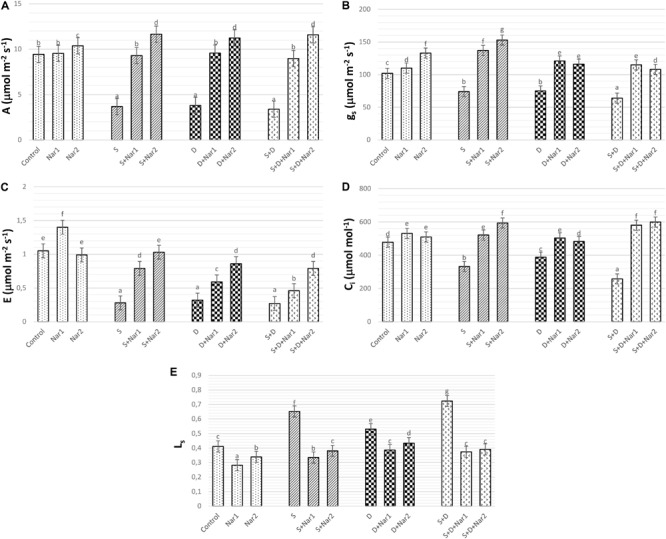
The effects of exogenously applied Nar (Nar1, 0.1 mM and Nar2, 0.4 mM) on the gas exchange parameters in bean leaves under alone or combination of salt (S, 100 mM NaCl) and PEG-induced osmotic (D, 10% PEG6000) stress. **(A)** Carbon assimilation rate (A), **(B)** stomatal conductance (g_s_), **(C)** transpiration rate (E), **(D)** intercellular CO_2_ concentration (C_i_), **(E)** stomatal limitation value (L_s_). For each group, vertical bars indicate ± SE and the different lowercase letters are significantly different (*p* < 0.05) values according to the Tukey test.

As well as the combined treatment, salt and osmotic stresses reduced A values, which reached the minimum levels at NaCl + PEG by 64.05% (Figure 5A). Similar trend was observed in g_s_ (Figure 5B), E (Figure 5C), and C_i_ (Figure 5D) values of stress-treated bean plants. The reduction rate of A, g_s_, E, and C_i_ was higher in the combination form of stresses. Also, these reductions in A, g_s_, E, and C_i_ were prevented by Nar treatments under stress conditions. On the other hand, after exposure to NaCl or PEG, the stomatal limitations resulted in 58.6 or 28.9% increase, respectively (Figure 5E). Both Nar applications caused a decline in L_s_ in response to stress or control conditions.

### The Effects of Nar on the Isozyme and/or Enzyme Compositions in Response to Stress

**FIGURE 6:**
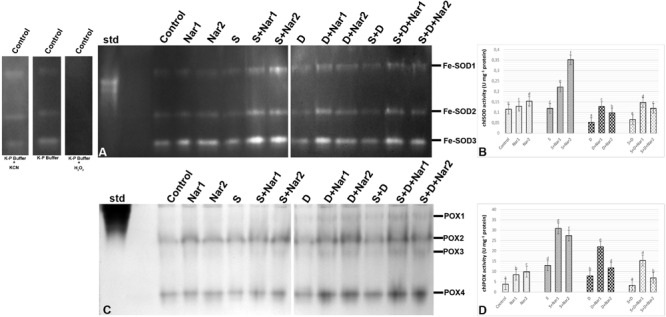
The effects of exogenously applied Nar (Nar1, 0.1 mM and Nar2, 0.4 mM) on some antioxidant enzyme activities in bean leaves under alone or combination of salt (S, 100 mM NaCl) and PEG-induced osmotic (D, 10% PEG6000) stress. **(A)** The relative band intensity of chloroplastic superoxide dismutase isoenzymes (SOD), **(B)** chloroplastic SOD activity, **(C)** the relative band intensity of different types of chloroplastic peroxidase isoenzymes (POX), **(D)** chloroplastic POX activity. Due to the high number of treatment groups and the low number of wells in the electrophoresis apparatus, the groups were divided into two as (i) C, Nar1, Nar2, S, S + Nar1, S + Nar2) and (ii) D, S, D + Nar1, D + Nar2, SD, SD + Nar1, SD + Nar2) and the gels were loaded to two different running modules at the same time. For the determination of SOD isozymes, before staining of SOD activity, the control group was preincubated with 50 mM potassium phosphate buffer (pH 7.8) alone, buffer plus 5 mM H_2_O_2_, or buffer plus 4 mM KCN. For each group, vertical bars indicate ± SE and the different lowercase letters are significantly different (*p* < 0.05) values according to the Tukey test.

As illustrated by Figure 6A, three SOD isozymes (Fe-SODs, Fe-SOD1-3) were detected by native page analysis in bean chloroplasts. However, Cu/Zn-SOD could not be identified. As compared to the control group, the chloroplastic SOD activity was reduced by PEG and NaCl + PEG (Figure 6B), depending on especially the intensities of Fe-SOD2 (Figure 6A). However, the plants treated with NaCl exhibited any effect in SOD activity. There was an increment in SOD activity of Nar-treated plants under stress. The induction in SOD (24.8%) was only observed the high Nar application under non-stress conditions.

Gel analysis revealed that four chloroplastic POX isozymes (POX1-4) were viewed in the evaluation of POX isozyme profiles (Figure 6C). The isozyme activity of POX of bean was higher by NaCl or PEG, providing the intensities of POX1-2. Interestingly, when applied a combination of both, no response was observed in POX activity (Figure 6D). In Nar plus stress-treated plants, the intensities of POX isoforms were stronger than stress treatments alone. This effect was related to the induced intensities of POX2-4 and the new defined isozyme, POX3. Exogenously applied Nar could maintain this induction produced by stress in POX under non-stress conditions as well.

**FIGURE 7:**
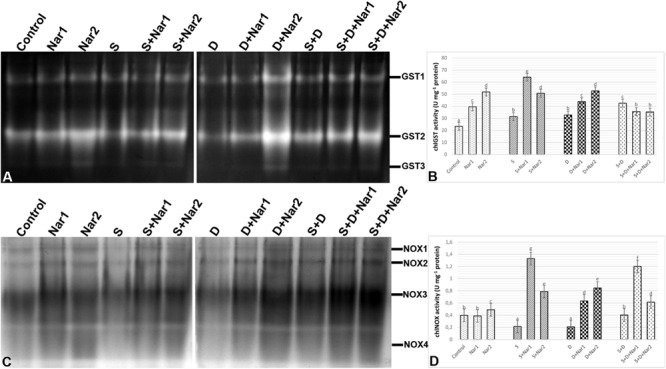
The effects of exogenously applied Nar (Nar1, 0.1 mM and Nar2, 0.4 mM) on some antioxidant enzyme activities in bean leaves under alone or combination of salt (S, 100 mM NaCl) and PEG-induced osmotic (D, 10% PEG6000) stress. **(A)** The relative band intensity of different types of chloroplastic glutathione S-transferase (GST), **(B)** chloroplastic GST activity, **(C)** the relative band intensity of different types of chloroplastic NADPH oxidase isoenzymes (NOX), **(D)** chloroplastic NOX activity. Due to the high number of treatment groups and the low number of wells in the electrophoresis apparatus, the groups were divided into two as (i) C, Nar1, Nar2, S, S + Nar1, S + Nar2) and (ii) D, S, D + Nar1, D + Nar2, SD, SD + Nar1, SD + Nar2) and the gels were loaded to two different running modules at the same time. For each group, vertical bars indicate ± SE and the different lowercase letters are significantly different (*p* < 0.05) values according to the Tukey test.

Quantification of the GST band intensities detected that three GST isozymes (GST1-3) revealed during experimental period (Figure 7A). The chloroplastic enzyme activity increased under all the stress treatments, as compared to the control group (Figure 7B). This induction of GST activity was related to the intensities of GST1-2 and GST1-2-3 in NaCl or PEG and NaCl + PEG-treated bean plants, respectively (Figure 7A). After NaCl treatment, Nar-treated plants had in induction in GST activity and it reached to the maximum level at S + Nar1, which was observed an enhancement by 2.03-fold. Similar trend was detected in the bean exposed to Nar together with PEG. On the other hand, chloroplastic GST activity of bean was lowered by Nar plus NaCl + PEG. Also, there was an enhancement in GST activity of Nar-treated plants under non-stress, as demonstrated by the stronger intensities of all GST isoforms.

As shown in Figure 7C, a total of four NOX isoenzymes was detected as NOX1-4 by native PAGE analysis. Chloroplastic NOX activity in bean was either unaffected or lower by the alone and the combined form of stress treatments (Figure 7D). However, bean leaves exposed to Nar and stress treatments exhibited the high NOX activity. This induction in chloroplastic NOX activity reached the maximum levels in Nar1 + NaCl-treated plants (6.2-fold). Although NOX3 isoform decreased in this treatment group (Nar1 + NaCl), the induction of the intensities of NOX1-2 was responsible for the response in activity. While 0.1 mM Nar alone created no remarkable effect on NOX activity, 0.4 mM Nar caused an increase in this enzyme compared to the control group (1.22-fold enhancement).

### The Effects of Nar on the Enzyme Activity Related to AsA-GSH Cycle in Response to Stress

**FIGURE 8:**
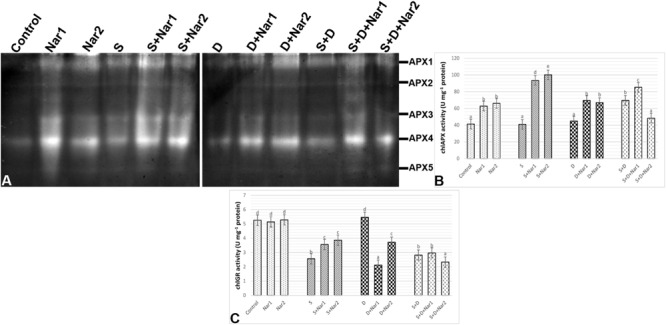
The effects of exogenously applied Nar (Nar1, 0.1 mM and Nar2, 0.4 mM) on some antioxidant enzyme activities in bean leaves under alone or combination of salt (S, 100 mM NaCl) and PEG-induced osmotic (D, 10% PEG6000) stress. **(A)** The relative band intensity of different types of chloroplastic ascorbate peroxidase isoenzymes (APX), **(B)** chloroplastic APX activity, **(C)** chloroplastic glutathione reductase (GR). Due to the high number of treatment groups and the low number of wells in the electrophoresis apparatus, the groups were divided into two as (i) C, Nar1, Nar2, S, S + Nar1, S + Nar2) and (ii) D, S, D + Nar1, D + Nar2, SD, SD + Nar1, SD + Nar2) and the gels were loaded to two different running modules at the same time. For each group, vertical bars indicate ± SE and the different lowercase letters are significantly different (*p* < 0.05) values according to the Tukey test.

Examination of chloroplastic APX isoenzymes in bean identified five isoforms (APX1-5) (Figure 8A). The bean leaves treated with NaCl or PEG had no change in the activity of APX enzyme. However, at both NaCl and PEG, a remarkable increase in APX was measured (66.2%) and especially APX1 and APX4 isozymes were responsible for this change. After Nar applications to the stress-applied bean, the activity levels of APX isozymes were induced throughout the experimental period. Also, this induction in APX activity was observed in only the 0.1 mM Nar-treated bean leaves under the combination form of stresses (Figure 8B), as provided the isozyme-staining pattern (Figure 8A). On the other hand, 0.1 and 0.4 mM Nar alone enhanced chloroplastic APX activity by 52.3% and 60.1% increment.

Except for PEG-treated plants, NaCl alone or together with PEG caused a reduction in chloroplastic GR activity (Figure 8C). Nar prevented this reduction in GR only in plants with NaCl. GR activity of the bean was either lower or unaffected by Nar + PEG and Nar+ the combined stress treatments. When compared to the control group, exogenously applied Nar created no remarkable effect on chloroplastic GR activity.

**FIGURE 9:**
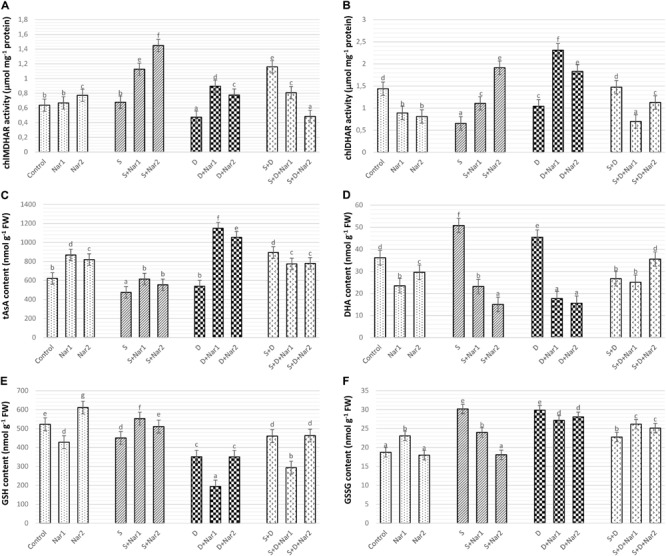
The effects of exogenously applied Nar (Nar1, 0.1 mM and Nar2, 0.4 mM) on some antioxidant enzyme activities related to AsA-GSH cycle in bean leaves under alone or combination of salt (S, 100 mM NaCl) and PEG-induced osmotic (D, 10% PEG6000) stress. **(A)** Chloroplastic monodehydroascorbate reductase activity (MDHAR), **(B)** chloroplastic dehydroascorbate reductase activity (DHAR), **(C)** chloroplastic ascorbate content (tAsA), **(D)** chloroplastic dehydroascorbate content (DHA), **(E)** chloroplastic glutathione content (GSH), **(F)** chloroplastic oxidized glutathione (GSSG). For each group, vertical bars indicate ± SE and the different lowercase letters are significantly different (*p* < 0.05) values according to the Tukey test.

There was a similar response between chloroplastic MDHAR (Figure 9A) and DHAR (Figure 9B) in stress-treated bean plants. Single stress treatments (NaCl or PEG) were reduced or did not change the activities of MDHAR and DHAR. However, in response to the combined stress treatment, chloroplastic MDHAR activity was induced by 82.1% (Figure 9A). After Nar application was added to NaCl or PEG stress-treated plants, the activities of MDHAR and DHAR were elevated in comparison with those of the stress-treated plants alone. On the other hand, the increased activity of these enzymes was not maintained with the applications of Nar under the combined stress treatments. While, there was no effect on MDHAR activity in chloroplasts of bean exposed to 0.1 mM Nar, the elevated activity of MDHAR was detected by 0.4 mM Nar (1.2-fold), as compared to the control group. When Nar was applied alone, a reduction in DHAR of bean chloroplasts was observed (Figure 9B).

Compared with control group, when the bean plants were exposed to stress, chloroplastic AsA content decreased or was similar to levels of control (Figure 9C). The combined stress-treated plants had an increase in AsA content. A remarkable increase in AsA was detected only in bean plants with Nar plus stress (NaCl or PEG). However, the induction in AsA was not maintained by Nar applications along with NaCl + PEG. In contrast to the results of AsA, single stress treatments caused an increment in DHA content which not observe in the combination form of stresses (Figure 9D). NaCl or PEG-induced increment in DHA content was not maintained by exogenously applied Nar. Besides, the chloroplasts of bean with S + D + Nar2 exhibited the increment in DHA content. Under non-stress conditions, Nar alone decreased DHA content as compared to the control group.

The contents of GSH (Figure 9E) or GSSG (Figure 9F) in chloroplasts of the bean decreased or increased for the experimental period when under stress, compared to the content level of the control group, respectively. While, only the bean leaves with Nar plus NaCl had high GSH content (Figure 9E), there was a reduction in NaCl or PEG (exception for PEG + NaCl) in GSSG content of Nar-treated bean chloroplasts (Figure 9F). Besides, when comparison to the control group, the maximum induction in GSH and GSSG was at Nar2 and Nar1 by 1.6-fold and 1.2-fold, respectively. Additionally, Nar applications minimized the risk of reducing GSH redox state [the ratio of GSH content to total glutathione (GSH + GSSG)] in response to NaCl stress.

### The Effects of Nar on H_2_O_2_ Content and Lipid Peroxidation Levels in Response to Stress

**FIGURE 10:**
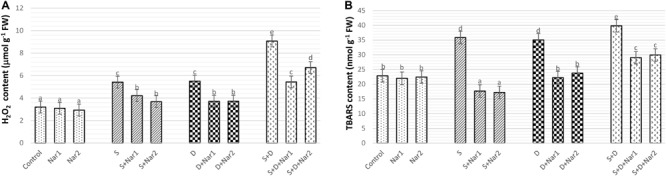
The effects of exogenously applied Nar (Nar1, 0.1 mM and Nar2, 0.4 mM) on the ROS content and lipid peroxidation in bean leaves under alone or combination of salt (S, 100 mM NaCl) and PEG-induced osmotic (D, 10% PEG6000) stress. **(A)** hydrogen peroxide (H_2_O_2_), **(B)** lipid peroxidation (TBARS content). For each group, vertical bars indicate ± SE and the different lowercase letters are significantly different (*p* < 0.05) values according to the Tukey test.

As detected in Figure 10A, H_2_O_2_ content gradually increased under stress treatments and reached the maximum levels (by 2.3-fold) in plants with NaCl plus PEG stress. While, Nar alone did not affect H_2_O_2_ content, after Nar treatments to the stress-treated bean plants, a decline in H_2_O_2_ content was detected.

Figure 10B reveals that stress treatments resulted in an induction in the levels of lipid peroxidation. The maximum rate of this increment in TBARS content was at NaCl + PEG by 74.1%. However, Nar alleviated the increased in TBARS content of both stress-treated plants. In contrast to H_2_O_2_ content, as compared to the control group, Nar applications had no increase in TBARS content.

## Discussion

### Physiological Parameters

Many reports revealed that stress reduces the osmotic potential (Ψ_Pi_) in plants (Sheldon et al., 2017). In the current study, when Ψ_Pi_ reached to more negative levels in bean under stress, the uptake of water or the efficiency of water use were reduced and then the water content (RWC) decreased. There was previous data where flavonoid levels can promote water content of rice seedlings under stress, as detected also in our study (Chutipaijit et al., 2009). In bean treated with Nar plus stress, Ψ_Pi_ reduced by NaCl and PEG was alleviated. This indicated that the induced levels of Ψ_Pi_ might help to conserve of RWC and to improve in hydration status, which was related to the higher levels of water content.

### The Photochemical Performance of PSII and the Transcript Levels of psbA and psbD Genes

To investigate for determination of the tolerance range of plants against to stress, the photochemical performance of PSII plays essential roles as an informative tool. As mentioned earlier, the responses of PSII are important in determining the differences on the photosynthetic machinery between salinity and osmotic stresses (Kalaji et al., 2018). The induction kinetics in fluorescence show the reduction of electron acceptors in electron transport system (ETS) (Gururani et al., 2015). A decline in F_v_/F_m_ and F_v_/F_o_ levels is reported in stress-treated plants (Kalaji et al., 2011). This reduction is evidence of the damage of reaction centers in PSII, the decrease in electron transfer rates at oxidizing site of PSII and the declined number of quanta absorbed per unit time (Mehta et al., 2010). In our study, NaCl and PEG stresses caused a decrease in electron flow to reaction center of PSII, by providing a decline in F_m_ (acceptor side of PSII) and the area (reduced plastoquinone pool size, data not shown). However, Nar had positive effects on F_v_/F_m_ and F_v_/F_o_ by promotion of this value reduced by stress. By increased levels in F_v_/F_m_, Nar was able to induce the capacity of the energy absorbed to the reaction center by PSII under dark-adapted conditions, as suggested by Shu et al. (2016). The possible reason behind Nar-triggered increase of F_v_/F_m_ and F_v_/F_o_ in bean might be repairment of the degraded photosynthetic pigments in the thylakoid membrane of chloroplasts or regulation of the reduction/re-oxidation levels of quinones. Phenomenological fluxes include energy flux in the excited cross section (CS) of the sample (ET_o_/CS and TR_o_/CS). After NaCl treatments, a decrease in ET_o_/CS_o_ caused the inactivation of reaction center. However, in stress-treated bean, Nar might regulate re-oxidation of reduced quinone through electron transfer (ET) over a CS of active and inactive reaction center (RC). In the current study, the lowered the electron transport per CS (TR_o_/CS) in stress-treated plants was alleviated by Nar application. NaCl and PEG-treated bean plants had a lower energy absorption by antenna pigments and energy trapping by RC. This is why there is a decrease in ET_o_/CS and TR_o_/CS under stress. However, the Nar-applied bean had the ability to protection of photosynthetic pigments such as chlorophyll-a. Similar phenomenon had been observed by Dolatabadian et al. (2012) that flavonoid genistein promoted photosynthesis rate by enhancing chlorophyll content in soybean exposed to salt stress.

NaCl and/or PEG caused an induction in DI_o_/CS, which was related to the levels of dissipated energy per leaf cross section. When DI_o_/CS was reduced by Nar application, the energy trapping efficiency in RC of PSII was higher than that of the stressed plants, which was also connected with the higher values of F_v_/F_m_. ABS/RC, ET_o_/RC and TR_o_/RC are the specific energy flux in membranes measured per the RC of sample. ABS/RC is the ratio of a number of absorbed energies by chlorophylls to the number of active RCs (Gururani et al., 2015). In the present study, this ratio was reduced under stress conditions (NaCl and/or PEG). This reduction was related to the decline in the amount of energy flow reaching to the RC of PSII and the antenna size of RCs. The stress-induced damage in PSII in this study was reversible after Nar application and the values reached to the higher levels than that of stress alone. ET_o_/RC and TR_o_/RC denote electron transfer and trap per active RC of PSII, respectively (Varghese et al., 2019). Trapping of an excitation by the RC results in the reduction of Q_A_ to Q_A_^–^ (Gururani et al., 2015). Under stress conditions, the overproduction of reduced Q_A_^–^ causes a damage in reaction center (Zhao et al., 2017). In the present study, stress decreased these parameters indicating a disruption in the transport and capture of electrons by photosynthetic systems, but this situation was successfully reversed by both of two different Nar application levels. Nar also protected bean leaves from photoinhibition by maintaining the reduced quinone pool. Exogenously applied Nar resulted in the positive effects on the forward electron transport rates by increasing ΨE_o_/(1-ΨE_o_) values. This recovery on ΨE_o_/(1-ΨE_o_) was related to the increased fluxes of absorption, trapping and electron transport in photosynthetic machinery (Rapacz et al., 2019). As well as the rate of biochemical reaction, both Nar treatments promoted the capacity of light reaction by the alleviated levels of ΦP_o_/(1-ΦP_o_). Besides, Nar promoted the reduction by stress on γRC/(1-γRC), which shows contribution of ET beyond reduced form of quinone. As contradistinction to the other parameters, DI_o_/RC was induced under stress treatments, as also suggested by Varghese et al. (2019). This situation has been regarded as an adaptive response for the dissipation of excess energy in a heat form based on the inactivation of PSII in photooxidative damage (Kalaji et al., 2014), in which electrons cannot be captured by RC of PSII. However, when Nar applied to the bean plants, this value did not need to increase, even it decreased in Nar alone and Nar + stress groups. The reason for this is that the excess energy caused stress-related damage did not occur in chloroplasts due to the exogenous application of Nar.

The other parameter of the OJIP test, the performance index (PI), indicates the vitality of sample under stress conditions and three critic steps in photosynthesis: absorption, trapping and electron transfer (Mehta et al., 2010; Varghese et al., 2019). In the present study, the reduced PI triggered by stress showed the decrement in density of the RC of PSII and this influence was greater under the combined stress treatments. Depending on efficiency of ΨE_o_/(1-ΨE_o_) and ΦP_o_/(1-ΦP_o_), Nar, in response to stress, encouraged the photosynthetic performance (PI_ABS_ and PI_total_) in bean chloroplasts. Our data are in contention with previous reports where measurement of diminished fluorescence in different stressed plants (Mehta et al., 2010; Lazar et al., 2013; Varghese et al., 2019). When all the structural and functional parameters mentioned above were evaluated, stress conditions (NaCl and/or PEG) reversibly resulted an inactivation in photosynthetic machinery in bean chloroplasts. However, Nar applications recovered the damage at the acceptor/donor side of PSII, the decrease in the pool size of reduced quinones or maintained the stability of PSII by enhancing the turnover of D1 protein (as detected in the increased transcription levels of psbA gene).

Because of containing several components which are necessary for the photochemical reactions, the D1 protein is a functional protein in PSII complex (Mukherjee, 2020). The regulation of gene expression related to the photosynthesis shows the tolerance against the stress conditions (Wang et al., 2017). One of them is psbA gene, which encodes the D1 protein and is responsible for the reproduction of impaired D1 protein by stress. The other gene is psbD which protects the stability of PSII complex and regulates D2 protein expression (Chen et al., 2016). In the present study, our data showed that NaCl and PEG disrupted the relative expression of psbA and psbD by triggering damage in PSII system, as shown by the results of F_v_/F_m_ and F_v_/F_o_. Similar conclusions were observed by Wang et al. (2018). However, both Nar applications protected transcription levels of psbA and psbD from the negative effects produced by NaCl or PEG, accelerating in the functional repair of PSII and assisting the synthesis of D2. This alleviation induced by Nar might be connected with newly synthesizing or repairing the impaired D1 protein.

### Gas Exchange

One of the factors associated with reduction of the photosynthetic rate in stress-treated plants is disruption on gas exchange parameters and water management. The water status in the plants is directly correlated with the changes in stomatal conductance (Wada et al., 2019). In the present study, the decline in g_s_ observed in salt and/or PEG-treated bean, signed to stomatal closure, which was accordance with significant declines in A, C_i_, and E. This data is consistent with report of Engineer et al. (2016). In the present study, the decline of A/C_i_ under NaCl and/or PEG showed that depending on decrease in A and C_i_, the assimilation values of CO_2_ in chloroplasts of NaCl or the PEG-treated bean might be influenced with the stomatal limitation, as evident by the increase in L_s_ (1- C_i_/C_a_). A similar observation was noted by Wang et al. (2018) in rice with stress. Nar applications could repair the restriction on photosynthetic regulation, as provided by a promotion of g_s_, A, C_i_, E, and a reduction in L_s_. Exogenously applied Nar in response to stress triggered stomatal opening by increasing g_s_ levels. This situation promoted transport of internal CO_2_ to chloroplasts (as proved increased C_i_) and carboxylation efficiency was alleviated. Under stress conditions, Nar applications could provide an increase in CO_2_ from the mesophyll (non-stomatal), as well as the increased entrance of CO_2_ through the stoma opening. C_i_/g_s_ is an important tool for identification of mesophyll efficiency and there is an opposite relationship between C_i_/g_s_ and mesophyll efficiency (Ramanjulu et al., 1998). In the current study, after individual treatments of NaCl or PEG to bean plants, both Nar applications caused a decline in C_i_/g_s_, which verified by the increased mesophyll efficiency, but that was not the case under the combined form of these stresses. Also, Nar applications in bean chloroplasts subjected to NaCl and/or PEG provided the recovery on the restriction in mesophyll and stomata, as evident from the results of A, E, g_s_, E, L_s_, and F_v_/F_m_.

### ROS Production and Chloroplastic Antioxidant Enzyme Activity

Because of the disruption in redox regulation in photosynthesis, the electrons with a high energy state of chlorophyll reduce molecular oxygen by forming singlet oxygen (^1^O_2_). Also, the thylakoidal electron transport components on the PSI side such as the Fe-S centers result in the reduction of oxygen by a reaction called the Mehler reaction (Cruz de Carvalho, 2008) thus forming superoxide and H_2_O_2_. SOD, converting superoxide anion radical to oxygen and H_2_O_2_, is the front-line enzyme against toxic accumulation of ROS (Zou et al., 2018). In the present study, despite no observation in response of chloroplastic SOD activity under NaCl and/or PEG, H_2_O_2_ content was induced. This result contradict data presented by Guan et al. (2017) who reported an increase in chloroplastic Cu/Zn-SOD of rice under salinity. Other than photosynthesis, photorespiration also produces ROS. During carbon assimilation, ribulose-1,5-bisphosphate is also oxidized with oxygen by the activity of enzyme Rubisco. The yielding glycolate is transported from chloroplasts to peroxisomes and H_2_O_2_ is generated (Hodges et al., 2016). Hence, a possible source of H_2_O_2_ is also the activation of some enzymes such as glycolate oxidases, glucose oxidases, amino-acid oxidases, sulfite oxidases and NOX (Wu et al., 2018). In the chloroplasts of bean, there was no contribution of NOX activity in H_2_O_2_ accumulation under stress. The produced H_2_O_2_ is eliminated by POX or APX in the so-called water-water cycle (Asada, 1999). In present study, POX activity increased only after individual application of NaCl or PEG, but, not under combined application of NaCl and PEG. Whereas, APX activity was induced only under NaCl plus PEG stress. Our observations are in line with that of Zhang et al. (2017) who reported increased activity of APX under NaCl + PEG in *Glycyrrhiza uralensis*, but not in NaCl alone-treated plants. The bean leaves showed more sensitivity to NaCl or PEG due to the reduction in AsA/DHA and GSH/GSSG ratio. In response to stress, the inadequate enzyme activity might also be related to the increased accumulation of H_2_O_2_. This was accordance with the higher TBARS content in bean chloroplasts under NaCl and/or PEG, as also reported by Wang et al. (2019).

Naringenin applications to NaCl and/or PEG-applied bean resulted in an increase of SOD activity, resulting in an increase in H_2_O_2_ content. Also, the induction of NOX activity also contributed to this increase. After Nar applications to bean with NaCl or PEG treatments, the differences were observed in the responses for scavenging of H_2_O_2_. When Nar was applied under NaCl, H_2_O_2_ produced by SOD or NOX was eliminated by the activities of POX and, APX, GR, MDHAR and DHAR which activated the AsA-GSH cycle. In response to NaCl stress, Nar increased chloroplastic DHA levels, which are converted from MDHA, depending on APX activity. Nar might trigger the transformation to AsA from MDHA via the activation of MDHAR. The induced DHA was regenerated to AsA by the catalysis of DHAR. The GSH regeneration required for the activity of DHAR was provided by GR which regenerates GSH and so, maintains the GSH pool (Shan et al., 2020). Under salt stress, the applications of Nar had direct capability to protect a high level of AsA/DHA. Therefore, Nar could maintain the pool of AsA and GSH under salt stress in bean chloroplasts by the regulation the AsA-GSH cycle. Also, the overexpressed enzymes presented in regeneration and biosynthesis of AsA and GSH display the enhanced the tolerance to stress (Li et al., 2012). The high ratio in GSH/GSSG and the elevated GSH content/redox state, which was detected in Nar-treated bean chloroplasts in response to salinity, showed that it could have a role in recycling of AsA and GSH by providing a redox state and reducing oxidative stress, as mentioned by Wang et al. (2013) and Garg and Singla (2015). Another enzyme, GST detoxifies toxic lipid peroxides and reduces dehydroascorbate with GSH-dependent reductase activity (DHAR) and so maintains the reductant pools such as AsA (Souri et al., 2020). In the current study, induced GST activity was continued by Nar applications with NaCl or PEG. However, under the combination of stresses, the increase in GST was not maintained via Nar applications. This result was inconsistent with the changes in AsA and GSH which interacts with ROS and abscisic acid in signaling pathway (Szalai et al., 2009). Hence, Nar might have promoted the signaling pathway via the increased GSH activity under salinity.

Exogenous addition of Nar together with PEG-induced osmotic stress to bean plants removed H_2_O_2_ accumulation through the activities of POX, APX, MDHAR, and DHAR. Like the responses to NaCl stress, Nar could be role in recycling of AsA through the induction of chloroplastic AsA content. Although DHAR converts to GSSG from GSH, it was not possible to regenerate GSH from GSSG due to the inactivity of GR. Therefore, Nar had no effect on GSH regeneration in bean chloroplasts treated with PEG. These results were consistent with the high ratio of AsA/DHA and the reduced GSH/GSSG and GSH redox state.

Interestingly, the same variation in this cycle was not observed in the combination with Nar and NaCl + PEG stress. No increases in the activities of GR, MDHAR, DHAR not only was observed in bean chloroplasts but also exhibited a reduction in the contents of AsA, DHA and GSH. So, the regeneration of both AsA and GSH contents was reduced by Nar applications under the combined form of these stresses. However, POX activity induced by Nar was successfully stimulated to remove of the toxic levels of H_2_O_2_ produced under NaCl + PEG. Our findings are in accordance with Agati et al. (2013) that the accumulation of H_2_O_2_ are eliminated by flavonoid-peroxidase reaction.

### Lipid Peroxidation

As a biomarker to measure the degree of damage, TBARS is important tool in plants treated with stress (Parvin et al., 2020) and TBARS is induced with salinity or osmotic stress in bean chloroplasts. When all this data was interpreted, Nar applications to stress-treated bean chloroplasts showed lower oxidative damage as indicated by lower H_2_O_2_ and TBARS levels. Similar observation was noted by Garg and Singla (2015), where Nar applications decreased both H_2_O_2_ and TBARS contents in *Cicer arietinum* under salt stress. Because of the decreasing amount of H_2_O_2_, Nar might trigger a signal role of H_2_O_2_ rather than its toxic effects.

## Conclusion

Our study confirms that Nar applications prevented inhibition on RWC, osmotic potential and photosynthetic efficiency (F_v_/F_m_, F_v_/F_o_, and F_o_/F_m_) effected by NaCl and/or PEG in bean chloroplasts. Nar could reverse the restriction on stomatal regulation, as evident by alleviation of g_s_, A, E and L_s_. Nar recovered the photosynthetic machinery by altering the stability of PSII [ABS/RC, ET_o_/RC and ΨE_o_/(1-ΨE_o_)] and regulation of dissipated energy levels absorbed energy by chlorophyll of all reaction center in PSII (DI_o_/CS_o_ and DI_o_/RC) and protection the damage at the acceptor/donor side of PSII. These regulations triggered by Nar led to a notable increase in the performance index (PI_ABS_) and the capacity of light reaction [ΦP_o_/(1-ΦP_o_)]. Both Nar applications (0.1 and 0.4 mM) protected from the negative effects produced by NaCl or PEG on transcription levels of psbA and psbD, accelerating in the functional repair of PSII and assisting the synthesis of D2. By regulating the antioxidant metabolism in chloroplasts of bean plants, Nar was able to control the toxic levels of ROS and TBARS produced by stress. Chloroplastic SOD activity reduced by stress in bean exposed to stress was increased by Nar. Since Nar exposure increased the activities of APX, GR, MDHAR, and DHAR as well as POX, Nar could maintain both AsA and GSH redox state in response to NaCl, as evident by enhancement in AsA/DHA and GSH/GSSG. Nar protected the bean chloroplasts by minimizing disturbances caused by salinity via the ascorbate and glutathione redox-based systems. Despite of the induction of MDHAR and DHAR under Nar plus PEG, Nar maintained the AsA regeneration, but not GSH recycling because of no induction in GR activity and the reduction in GSH/GSSG and GSH redox state. Consequently, Nar protected bean chloroplasts by minimizing disturbances caused by NaCl or PEG exposure via the AsA or GSH redox-based systems and POX activity.

## Data Availability Statement

All datasets generated for this study are included in the article/Supplementary Material.

## Author Contributions

EY, CO-K, MK, and IT conceived and designed the research. CO-K and EY conducted the experiments. CO-K, EY, and IT analyzed the data. CO-K, EY, and IT wrote the manuscript. All authors read and approved the manuscript.

## Conflict of Interest

The authors declare that the research was conducted in the absence of any commercial or financial relationships that could be construed as a potential conflict of interest.
