# Supplementary material for: Flavonoid Naringenin Alleviates Short-Term Osmotic and Salinity Stresses Through Regulating Photosynthetic Machinery and Chloroplastic Antioxidant Metabolism in Phaseolus vulgaris
Source: Front Plant Sci. 2020 Jun 3;11:682. doi: 10.3389/fpls.2020.00682 (PMC7283533; doi:10.3389/fpls.2020.00682)
Supplement: Supplementary file 1 [file Table_1.doc]

**Supplementary Table S1**. Definition of treatments used in the current study.

| **Number of groups** | **Treatments** | **Definition** |
| --- | --- | --- |
| 1 | Control | Normal conditions without stress or Nar |
| 2 | Nar1 | 0.1 mM Nar application alone without stress treatments |
| 3 | Nar2 | 0.4 mM Nar treatment alone without stress treatments |
| 4 | S | Salt treatment (100 mM NaCl) alone without Nar applications |
| 5 | S+Nar1 | Salt treatment (100 mM NaCl) together with 0.1 mM Nar application |
| 6 | S+Nar2 | Salt treatment (100 mM NaCl) together with 0.4 mM Nar application |
| 7 | D | Osmotic stress (10% PEG-6000) alone without Nar applications |
| 8 | D+Nar1 | Osmotic stress (10% PEG-6000) together with 0.1 mM Nar application |
| 9 | D+Nar2 | Osmotic stress (10% PEG-6000) together with 0.4 mM Nar application |
| 10 | S+D | Salt treatment (100 mM NaCl) together with osmotic stress (10% PEG-6000) |
| 11 | S+D+Nar1 | Salt treatment (100 mM NaCl) together with osmotic stress (10% PEG-6000) under 0.1 mM Nar application |
| 12 | S+D+Nar2 | Salt treatment (100 mM NaCl) together with osmotic stress (10% PEG-6000) under 0.4 mM Nar application |
