# Supplementary material for: Flavonoid Naringenin Alleviates Short-Term Osmotic and Salinity Stresses Through Regulating Photosynthetic Machinery and Chloroplastic Antioxidant Metabolism in Phaseolus vulgaris
Source: Front Plant Sci. 2020 Jun 3;11:682. doi: 10.3389/fpls.2020.00682 (PMC7283533; doi:10.3389/fpls.2020.00682)
Supplement: Supplementary file 2 [file Table_2.DOC]

**Supplementary Table S2**. Gene accession numbers and primer sequences of the genes described in this study. The experiments were identified for qRT-PCR assays with the StepOnePlus™ Real-Time PCR System (Applied Biosystems™) and the data was analyzed with StepOne™ Software version 2.2.2. qRT-PCR conditions were as follows: 95°C for 30 s and 40 cycles of 95°C for 5 s and 60°C for 30 s. The amplicons specificities were detected by melting curves analysis (60 to 95°C) after 40 PCR cycles.

| **Accession No** | **Gene name** | **Forward** | **Reverse** |
| --- | --- | --- | --- |
| 4961753 | psbA- photosystem II protein D1 | 5´-TGCTACATGGGTCGTGAATG-3´ | 5´-GCTGCAACAGGAGCTGAATA-3´ |
| 4961781 | psbD photosystem II protein D2 | 5´-TGGGAGTTGCTGGTGTATTG-3´ | 5´-CTTCAGCTTGGGTTGGGTTA-3´ |
| 820385 | Actin11 | 5´-TGCATACGTTGGTGATGAGG-3´ | 5´-AGCCTTGGGGTTAAGAGGAG-3´ |
